# Daikenchuto (TU‐100) shapes gut microbiota architecture and increases the production of ginsenoside metabolite compound K

**DOI:** 10.1002/prp2.215

**Published:** 2016-02-10

**Authors:** Takumu Hasebe, Nobuhiro Ueno, Mark W. Musch, Anuradha Nadimpalli, Atsushi Kaneko, Noriko Kaifuchi, Junko Watanabe, Masahiro Yamamoto, Toru Kono, Yuhei Inaba, Mikihiro Fujiya, Yutaka Kohgo, Eugene B. Chang

**Affiliations:** ^1^ Department of MedicineInflammatory Bowel Disease CenterThe University of ChicagoChicagoIllinois; ^2^ Division of Gastroenterology and Hematology/OncologyDepartment of MedicineAsahikawa Medical UniversityAsahikawaHokkaidoJapan; ^3^ Tsumura Research LaboratoriesTsumura & Co., AmiIbarakiJapan; ^4^ Faculty of Pharmaceutical SciencesHokkaido UniversitySapporoHokkaidoJapan; ^5^ Center for Clinical and Biomedical ResearchSapporo Higashi Tokushukai HospitalSapporoHokkaidoJapan

**Keywords:** Bacterial metabolism, bioavailability, Daikenchuto, microbiome, traditional Japanese medicine

## Abstract

Many pharmaceutical agents not only require microbial metabolism for increased bioavailability and bioactivity, but also have direct effects on gut microbial assemblage and function. We examined the possibility that these actions are not mutually exclusive and may be mutually reinforcing in ways that enhance long‐term of these agents. Daikenchuto, TU‐100, is a traditional Japanese medicine containing ginseng. Conversion of the ginsenoside Rb1 (Rb1) to bioactive compound K (CK) requires bacterial metabolism. Diet‐incorporated TU‐100 was administered to mice over a period of several weeks. T‐RFLP and 454 pyrosequencing were performed to analyze the time‐dependent effects on fecal microbial membership. Fecal microbial capacity to metabolize Rb1 to CK was measured by adding TU‐100 or ginseng to stool samples to assess the generation of bioactive metabolites. Levels of metabolized TU‐100 components in plasma and in stool samples were measured by LC‐MS/MS. Cecal and stool short‐chain fatty acids were measured by GC‐MS. Dietary administration of TU‐100 for 28 days altered the gut microbiota, increasing several bacteria genera including members of *Clostridia* and *Lactococcus lactis*. Progressive capacity of microbiota to convert Rb1 to CK was observed over the 28 days administration of dietary TU‐100. Concomitantly with these changes, increases in all SCFA were observed in cecal contents and in acetate and butyrate content of the stool. Chronic consumption of dietary TU‐100 promotes changes in gut microbiota enhancing metabolic capacity of TU‐100 and increased bioavailability. We believe these findings have broad implications in optimizing the efficacy of natural compounds that depend on microbial bioconversion in general.

**Abbreviations**
CK: compound K
GF: germ free
Kampo: natural product, herbal medicine, complementary and alternative medicine
LC‐MS/MS: liquid chromatography–tandem mass spectrometry
PCA: principal component analysis
Rb1: ginsenoside Rb1
SPF: specific pathogen free
T‐RFLP: terminal restriction fragment length polymorphism
TU‐100: Daikenchuto
Treg: regulatory T

## Introduction

Enteric microbiota exerts diverse and profound effects on the physiology of the host. Considerable evidence strongly suggests that dietary intake, particularly a shift to a westernized diet, contributes to development and progression of diseases such as obesity, metabolic liver disorders, rheumatoid arthritis, and inflammatory bowel disease (Huang et al. 2013). Many factors influence the composition of the intestinal microbiome including dietary components such as lipid and carbohydrate, as well as medical compounds that increase or decrease the survival and growth of specific types of bacteria. Bacteria have diverse metabolic activities and are required for metabolism of many endogenous and xenobiotic substances prior to intestinal absorption, increasing bioavailability. One xenobiotic compound where bacterial metabolism is essential for intestinal absorption is the ginseng saponin, ginsenoside Rb1 (Rb1) (Wang et al. 2011). Ginseng is a family of plants and various species that synthesize many chemicals, including heterocyclic, dammarane ring compounds termed ginseng saponins. Rb1 is a major saponin of all ginseng, produced at high levels by Wisconsin (*Panax quinquefolius*) and Asian ginseng (*Panax ginseng*), but present in all of the genus *Panax* members. Roots of most *Panax* are fleshy and have been used in traditional medical uses for thousands of years and contain the high levels of ginsenoside Rb1.

Daikenchuto (TU‐100), a pharmaceutical grade Japanese traditional (KAMPO) medicine, has been approved by the government regulatory health agency and widely prescribed in Japan for the treatment of postoperative paralytic ileus and ischemic intestinal disorders (Kono et al. 2009). Several double blind placebo‐controlled trials on the patients with postoperative ileus, Crohn's disease, and irritable bowel syndrome are currently underway in the United States and Japan (Manabe et al. 2010; Iturrino et al. 2013; Okada et al. 2013; Shimada et al. 2015). These clinical studies are tests for clinical pharmacology and early phase II investigation. Although Manabe et al. has shown a significant enhancement of colonic emptying in healthy volunteers (Manabe et al. 2010), the efficacy of TU‐100 in GI disease patients is presently being established. TU‐100 is an extract powder prepared from a mixture of processed ginger, Japanese pepper (*Zanthoxilum* fruit), and *P. ginseng*. Ginger root has distinct synthetic capabilities and produces many compounds, including gingerols and shogaols. Japanese pepper also produces diverse chemicals most studied being the hydroxysanshools. These compounds may act in complementary and synergistic ways (Kono et al. 2015). The rapid and direct action of these ingredients on intestinal epithelial cells, lymphocytes, enteric nerves, and smooth muscle cells are involved in TU‐100's vasodilatory and prokinetic effects (Satoh et al. 2001; Murata et al. 2002; Kito and Suzuki 2006; Tokita et al. 2007, 2011; Kono et al. 2011; Kono et al. 2013; Ueno et al. 2014). TU‐100 is frequently used chronically, especially for alleviation of abdominal bloating. TU‐100 shortened the postoperative periods until the first flatus in the patients with hepatic resection (Nishi et al. 2012) and laparoscopic colorectal resection (Yoshikawa et al. 2012), and suppressed elevation of blood ammonia level after partial hepatectomy (Kaiho et al. 2005). A major source of both gas (methane and hydrogen sulfide) and ammonia is intestinal microbiota (Sahakian et al. 2010; Dhiman 2013), which suggests the change in microbial population induced by TU‐100 treatment may be involved in the mechanism of action of the medicine. Research cited on TU‐100 has focused on intestinal tract effects as historically these are the symptoms for which the TU‐100 formulation was used. Intestinal bloating, cramping, and flatulence are indications for TU‐100 use. The ability of TU‐100 compounds to stimulate motility has been most investigated. The green pepper sanshools activate TRPV and TRPA channels that increase adrenomedullin (and other calcitonin gene‐related peptides) production by intestinal epithelial cells which increase intestinal motility. Motility effects of TU‐100 are also mediated by ginger and ginseng compounds. Gingerols and shogaols (Yamahara et al. 1990) and ginsenosides (Hashimoto et al. 2003; Kim et al. 2007) accelerate intestinal transit. The use of TU‐100 for bloating and motility has prompted its use for irritable bowel syndrome. TU‐100 effects on intestinal gases and ammonia have, however, received less mechanistic investigation.

The present studies examine the time‐dependent effects of TU‐100 on the gut microbiota, demonstrating that dietary ingestion of TU‐100 is associated with skewing of the gut microbial membership and function to increase the bioavailability of its bioactive metabolites. We believe these findings have broad implications to how the effectiveness of natural compounds like TU‐100 can be optimized through manipulation of the gut microbiome.

## Materials and Methods

### Mouse studies and ethics statement

All animal work was approved by the University of Chicago Institutional Animal Care and Use Committee (IACUC protocol 72101). C57Bl6/J mice were bred in house for all studies. Mice were from 6–12 weeks of age and both genders were used. Mice were sacrificed using CO_2_ followed by cervical dislocation as approved by IACUC.

### Compliance with design and statistical analysis requirements

More than five mice were used for all experimental groups randomly, no inclusion or exclusion criteria used. Samples were run in singlicate, each mouse representing *n* = 1. Data are shown as mean ± SD. A univariate analysis was conducted with the unpaired Student's *t* test to determine significance, considered significant at *P* < 0.05.

### TU‐100 components

TU‐100 was obtained as powder from Tsumura & Co. (Ami, Ibaraki, Japan). TU‐100 was included in mouse diet AIN‐76A, a defined diet (Harlan Teklad, Madison, WI; CA.170481) at 15 g TU‐100/kg diet (1.5%wt/wt) (Harlan Teklad; TD.110333). The dosage of TU‐100 was determined by the doses used in the previous studies using mice (Kaneko et al. 2013; Kono et al. 2013; Ueno et al. 2014; Watanabe et al. 2015). In these studies, TU‐100 exerted pharmacological effects in mice similar to its clinical efficacy in humans and blood concentrations of its major ingredients were in range to human data after ingestion of TU‐100.

### Collection of stool samples

To study temporal changes in the intestinal microbiome, stool samples were collected before switching mice from standard chow to AIN‐76A. They were allowed for 1 week equilibration on this diet and stool taken at designated times after the diet change.

### DNA extraction

For DNA extraction, 50 mg stool samples were dissolved in 1 mL lysis buffer (50 mmol/L Tris [pH 7.4], 100 mmol/L EDTA [pH 8.0], 400 mmol/L NaCl, 0.5% SDS; salts and chemical reagents from Thermo Fisher, Itasca, IL) containing 20 *μ*L proteinase K (20 mg/mL). About 500 *μ*L slurry of 0.1 mm diameter zirconia/silica beads (BioSpec Products, Bartlesville, OK) were added into the extraction tubes and a Mini‐Beadbeater‐8k Cell Disrupter (BioSpec) used to lyse the bacteria. After overnight incubation at 55°C, standard DNA extraction with phenol:chloroform:isoamyl alcohol and precipitation with ethanol were performed. Isolated DNA was dissolved in nuclease‐free water and stored at −80°C.

### Terminal restriction fragment length polymorphism (T‐RFLP) analysis

16S rRNA gene sequences were amplified from DNA samples using broad‐range primers 8F (5′‐AGAGTTTGATCCTGGCTCAG‐3′) labeled with 5′ carboxyfluorescein (5‐FAM) and 1492R (5′‐GGTTACCTTGTTACGACTT‐3′) for the conserved 16S bacterial domain. PCR reactions were performed for 30 cycles using TaKaRa high‐fidelity ExTaq (TaKaRa Mirus Bio, Madison, WI) with an annealing temperature of 58°C. An aliquot of the PCR reaction was analyzed by electrophoresis and the remainder precipitated. FAM‐labeled PCR products were then digested by restriction enzyme Msp I (New England Biolabs, Beverly, MA), dialyzed by placing on 0.025 *μ*m VSWP nitrocellulose filters (Millipore, Billerica, MA) on water for 7 min, removed and mixed with GeneScan‐500 size standard (Applied Biosystems, Foster City, CA), and analyzed in the Gene Scan mode of the Applied Biosystems 3730 sequencer in the University of Chicago Comprehensive Cancer Center Sequencing Facility.

### Principal coordinate analysis

Online software for processing and analysis of T‐RFLP data, T‐REX (trex.biohpc.org/) was used.

### 454 DNA sequencing

As T‐RFLP does not provide taxonomical information, samples were selected and analyzed by 454 pyrosequencing (V3–V5 region). DNA sequencing was performed at Research and Testing Laboratories (Lubbock, TX). Sequences were trimmed and aligned with the online database Ribosomal Database Project (RDP, Michigan State University, Lansing, MI, USA). 16S rRNA gene sequences were classified at a number of levels, phylum to genus or species levels.

### Measurement of bacterial metabolic ability of TU‐100 and ginseng ingredients

Stools were collected from mice fed AIN‐76A diet with or without 1.5% TU‐100 diet for 28 days. Stool was weighed and suspended at 100 mg/mL in 50 mM NaHPO_4_, pH 7. Thirty microliters of suspension were mixed with 240 *μ*L phosphate buffer and 30 *μ*L TU‐100 solution (20 mg/mL), ginseng solution (6 mg/mL) or distilled water as a vehicle and incubated at 37°C for 24 h in room temperature in a water bath. Reactions were extracted into 1500 *μ*L butanol (3 times 500 *μ*L each) to ensure complete extraction of TU‐100 or ginseng components and their metabolites and dried under nitrogen. Dried components were resuspended in 200 *μ*L methanol and concentrations of ginsenosides (Rb1, Rg1, Rf, Rh1, F1, and CK), sanshools, hydroxy‐*α*‐sanshool (HAS), and hydroxy‐*β*‐sanshool (HBS), and shogaols, [6]‐shogaol (6S), and [10]‐shogaol (10S) were analyzed by LC‐MS/MS. Details of preparation of standard solutions and quality control samples, instrumentation, analytical conditions, and validation of the analytical method for TU‐100 ingredients were described in a previous study (Iwabu et al. 2010). Diagrams of metabolic pathways of major ginseng ingredients are shown in Figures S1A and B. Amounts of each component in each reaction were analyzed by subtracting the amount of components contained in stools and incubated overnight without added TU‐100 or ginseng to determine levels present in the stool.

### Short‐chain fatty acid measurements

Before sacrifice, stool was collected and processed from individual mice using the same procedure used for cecal contents. Stool or cecal contents were weighed (7–15 mg of either source was used) and 600 *μ*L water added. Samples were resuspended well, allowed to sit for 5 min and solid material pelleted (13,000*g* for 20 sec at room temperature). Then, 500 *μ*L were removed and transferred to a fresh tube, 100 *μ*L of 50% sulfuric acid was added. This mixture was extracted three times with 500 *μ*L diethyl ether, centrifuging at 13,000*g* for 20 sec to separate phases. Ether extracts were combined and mixed. One microliter was removed and reacted with 250 *μ*L solution of MTB‐STFA (Sigma, St. Louis MO). Samples (5 *μ*L) were analyzed on a Varian Saturn 2000 GC/MS‐MS within 1 day. Standard curves of acetate, propionate, and butyrate (Sigma Aldrich, Milwaukee, WI) in water were diluted in saline, extracted, and derivatized to obtain a standard curve.

## Results

### Alteration of microbiome in TU‐100‐fed mice stools

**Figure 1:**
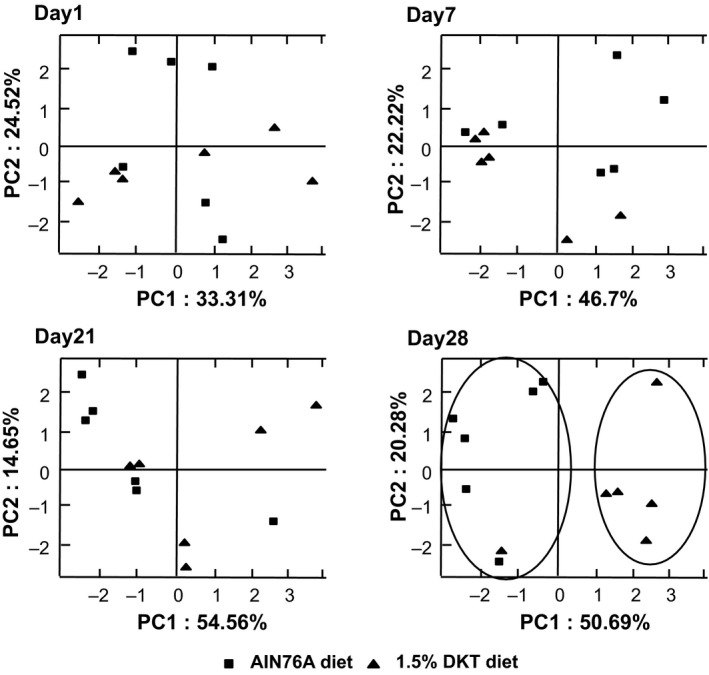
Clustering of fecal T‐RFLP (terminal restriction fragment length polymorphism) samples based on dietary treatment. Principal coordinate analysis (PCA) was performed based on the weighted UniFrac distance matrix generated from T‐RFLP profile in samples from mice at days 1, 7, 14, and 28 of the TU‐100 containing diet with and without TU‐100. Clustering was observed only after 28 days on dietary TU‐100. The *x*‐axis is the primary coordinate and *y*‐axis the secondary coordinate. Axis numbering represents the relative distance between samples based on the weighted UniFrac distance matrix. *n* = 6 mice in each group.

Fecal samples collected from mice with or without dietary TU‐100 over 1 month were first examined by T‐RFLP analysis. Electropherograms of Msp I‐digested 16S rRNA gene profiles of mouse stool DNA demonstrated that the microbial profiles of TU‐100‐fed mice became distinct from those of the mice fed diet without TU‐100 after 28 day treatment (Fig. 1). In order to obtain taxonomical information on the microbial changes, 16S rRNA genes in stool DNA were analyzed by pyrosequencing (the list of examined microbiota is shown in Table S1). Phylum level profiling shown in Table 1 demonstrated no significant changes in TU‐100‐treated mice. At the genus level, however, a significant increase is observed in a number of genera including *Lactococcus* sp. (Table 2) as well as four species of *Clostridium* subcluster XIVa (*Clostridium clostridioforme*,* Clostridium populeti*,* Roseburia intestinalis*, and *Eubacterium hallii*) (Collins et al. 1994; Barcenilla et al. 2000) known to produce short‐chain fatty acids, including butyrate and acetate. Other bacteria listed in Table 2 increased by TU‐100 are known producers of short‐chain fatty acids including *Clostridium thermocellum* (Weimer and Zeikus 1977), *Clostridium asparagiforme* (Mohan et al. 2006), *Bryantella formatexigens* (Rey et al. 2010) that produce acetate, *Clostridium propionicum* which produces propionate (Liu et al. 2014), and *Roseburia intestinalis* and *Eubacterium hallii* that produce butyrate (Munoz‐Tamayo et al. 2011; Van den Abbeele et al. 2013).

**Table 1:** The effect of TU‐100 ingestion on the composition of fecal bacterial phyla

Phylum name	Population (% of total sequences)		*P* value
	AIN76A diet	1.5% TU‐100 diet	
*Bacteroidetes*	26.31 ± 6.54	20.81 ± 4.27	0.1662
*Firmicutes*	71.28 ± 5.11	77.84 ± 4.21	0.0777
*Proteobacteria*	1.01 ± 0.38	0.64 ± 0.36	0.1866
*Tenericutes*	0.03 ± 0.05	0.09 ± 0.06	0.2014
*Acidobacteria*	0.27 ± 0.17	0.36 ± 0.15	0.4379
*Deferribacteres*	1.10 ± 1.31	0.26 ± 0.24	0.1471

Data represent means ± SD (*n* = 3–6). Statistical analysis was performed by unpaired Student's *t* test.

**Table 2:** The effect of TU‐100 ingestion on the composition of fecal bacterial genera

Name	Phylum	Family	Population (% of total sequences)		*P* value
			AIN76A diet	1.5% TU‐100 diet	
*Clostridium* sp.	*Firmicutes*	*Clostridiaceae*	19.118 ± 3.982	27.219 ± 4.512	0.0437
*Clostridium clostridioforme*	*Firmicutes*	*Clostridiaceae*	0.017 ± 0.019	0.104 ± 0.058	0.0323
*Clostridium populeti*	*Firmicutes*	*Clostridiaceae*	0.140 ± 0.011	0.525 ± 0.197	0.0031
*Clostridium sulfidigenes*	*Firmicutes*	*Clostridiaceae*	0.033 ± 0.037	0.159 ± 0.121	0.0417
*Clostridium thermocellum*	*Firmicutes*	*Clostridiaceae*	0.000 ± 0.000	0.025 ± 0.021	0.0454
*Clostridium asparagiforme*	*Firmicutes*	*Clostridiaceae*	0.041 ± 0.038	0.146 ± 0.068	0.0228
*Bryantella formatexigens*	*Firmicutes*	*Clostridiaceae*	0.976 ± 0.290	0.374 ± 0.266	0.0427
*Clostridium propionicum*	*Firmicutes*	*Clostridiaceae*	0.004 ± 0.007	0.028 ± 0.014	0.0254
*Lactococcus* sp.	*Firmicutes*	*Streptococcaceae*	1.176 ± 0.137	4.770 ± 2.602	0.0308
*Lactococcus lactis*	*Firmicutes*	*Streptococcaceae*	0.387 ± 0.116	1.153 ± 0.612	0.0195
*Peptococcus* sp.	*Firmicutes*	*Peptococcaceae*	0.017 ± 0.019	0.084 ± 0.041	0.0260
*Staphylococcus* sp.	*Firmicutes*	*Staphylococcaceae*	0.000 ± 0.000	0.084 ± 0.007	0.0024
*Roseburia intestinalis*	*Firmicutes*	*Lachnospiraceae*	0.358 ± 0.251	1.616 ± 0.861	0.0201
*Eubacterium hallii*	*Firmicutes*	*Eubacteriaceae*	0.000 ± 0.000	0.022 ± 0.020	0.0489
*Alistipes* sp.	*Bacteroidetes*	*Rikenellaceae*	0.120 ± 0.074	0.684 ± 0.288	0.0039
*Alistipes shahii*	*Bacteroidetes*	*Rikenellaceae*	0.000 ± 0.000	0.044 ± 0.020	0.0122
*Anaerophaga* sp.	*Bacteroidetes*	*Rikenellaceae*	0.000 ± 0.000	0.039 ± 0.035	0.0292

Data represent means ± SD (*n* = 5–6). Statistical analysis was performed by unpaired Student's *t* test.

### Metabolic activity of stool microbiota for TU‐100 ingredients

**Figure 2:**
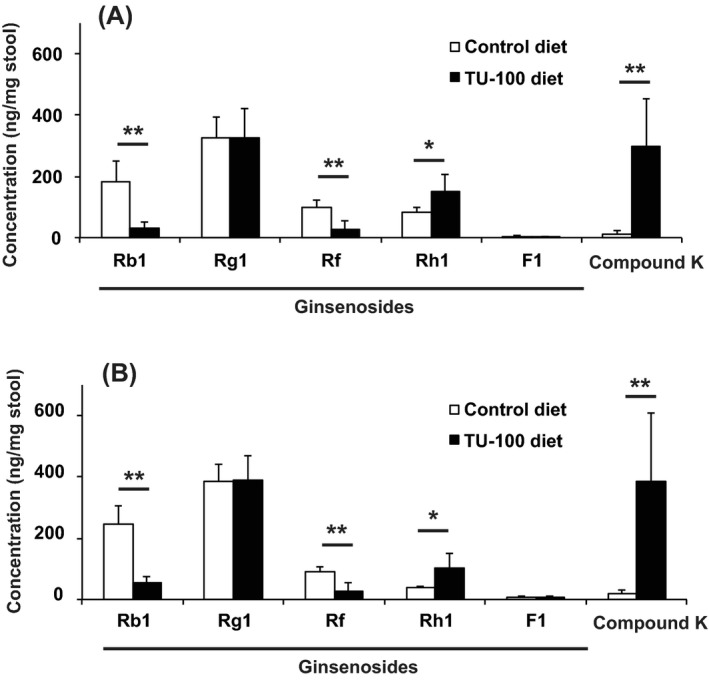
Assessment of stool sample capacity to bioconvert TU‐100 and its components. Solutions of TU‐100 (A) or ginseng extract (B) were added to the stool prepared from the mice treated for 28 days with TU‐100 diet (closed column) and control diet without TU‐100 (open column). After 24 h incubation at 37°C, ingredients and metabolites were analyzed by LC‐MS/MS as described in Materials and Methods section. The values before incubation were subtracted from those after incubation and expressed as means ± SD. *P < 0.05, **P < 0.01 (*n* = 6).

We analyzed the activity of the fecal microbiome to metabolize TU‐100 compounds. A suspension of fecal sample was added to extracts of TU‐100 or ginseng and incubated overnight at 37°C. The ingredients and metabolites before and after addition of the fecal suspension were analyzed by LC‐MS/MS (Fig. 2A and B). Rb1 and ginsenoside Rf (Rf) significantly decreased and CK (compound k) and ginsenoside Rh (Rh) significantly increased. Other major ingredients and metabolites, specifically ginsenoside Rg1 (Rg1) and F1 from ginseng, were not affected by the incubation with fecal suspension. HAS and HBS from Japanese pepper, and 6S and 10S from processed ginger, were also unaffected (data not shown).

### Pharmacokinetics of TU‐100 ingredients

**Figure 3:**
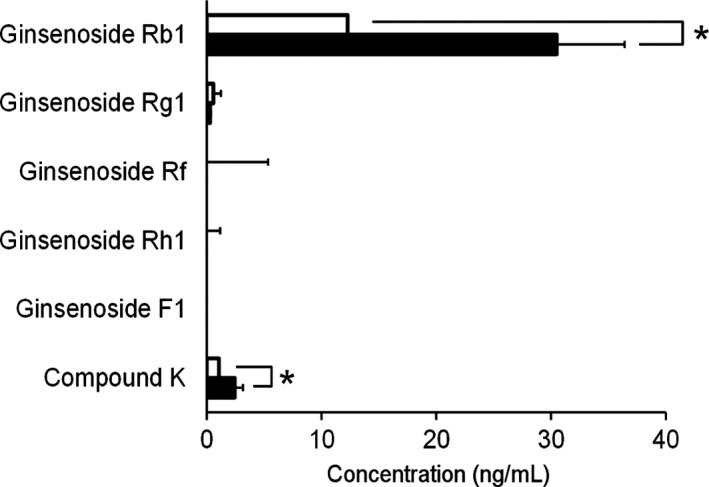
Plasma concentrations of TU‐100 ingredients and metabolites in mice. Mice were treated with TU‐100 for 1 day (open bar) or 28 days (closed bar). Plasma samples were analyzed by LC‐MS/MS as described in Materials and Methods section. Data represent means ± SD. *P < = 0.05 (*n* = 6).

In order to investigate whether the difference in the metabolic capacity of microbiota affects pharmacokinetics of TU‐100's active ingredients, we examined the blood concentrations of ginseng ingredients and its metabolites after 28‐day ingestion of TU‐100 (Fig. 3). We compared these result to those obtained from mice that had ingested TU‐100 for only 1 day. In blood collected from 28‐day TU‐100‐treated mice, concentrations of CK and Rb1 were significantly higher than in mice with ingestion for 1 day. To assess pharmacokinetics before any dietary exposure, a single oral administration of TU‐100 to SPF (specific pathogen free) and GF (germ free) mice was performed (Fig. S2). Similar to previous studies in humans (Munekage et al. 2011), in SPF mice Rb1 was slowly absorbed, peaked at 2 h, and decreased slowly. After 24 h, over 80% of the peak Rb1 amount was still present in the blood. Similar results were obtained for Rb1 in GF mice demonstrating that microbial action was not required for Rb1 absorption. In SPF mice, CK was detected starting at 2 h and increasing thereafter. In GF mice, CK was not detected in any mice at any time point, demonstrating the requirement for microbial conversion.

### Dietary TU‐100 increases short‐chain fatty acid generation

**Figure 4:**
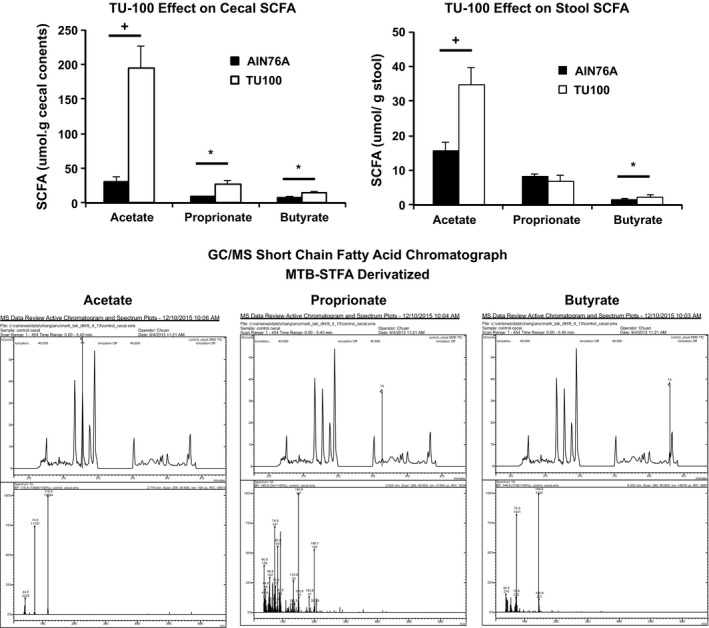
Cecal and stool short‐chain fatty acids. Mice were fed TU‐100 for 28 days and cecal contents and stool from the same mice were analyzed for short‐chain fatty acids as described in Materials and Methods section by GC‐MS. Data represent means ± SE. *P < 0.05, +*P* < 0.01 (*n* = 7). The bottom set of panels is analysis of a cecal sample from a mouse fed diet without TU‐100. The chromatographs are marked with vertical lines labeled 1A for acetate (left), propionate (middle), and butyrate (right). The m/z for these peaks is presented below and confirmed identification as these short‐chain fatty acids.

Many intestinal bacteria produce short‐chain fatty acids, acetate, propionate, or butyrate from undigested complex carbohydrates. Additionally, bacteria produced compounds like lactate that can be converted by other microbes into SCFA. Mice were fed diet with or without TU‐100 for 28 days and cecal contents and stool from the same mouse analyzed for short‐chain fatty acids. As shown in Figure 4, cecal short‐chain fatty acids (top left panel) were much greater than levels in stool (top right panel). Presumably, this represented absorption of these fatty acids as they transit through the host large intestine. Dietary TU‐100 increased all short‐chain fatty acids in cecal contents and acetate and butyrate in stool. The bottom set of panels is a representative gas chromatograph for the system used. This sample is a cecal sample from a mouse fed AIN76A. The top panels for each are time courses of eluted compounds and acetate is marked on the left, propionate in the middle, and butyrate on the right by a vertical line labeled 1 A by the mass spectrometer. The bottom panels are the m/z values for this 1 A labeled peak above and m/z for acetate is 116, propionate 133, and butyrate 145. Standards were run as well as m/z values to confirm identification.

## Discussion

The present study demonstrates that the clinical efficacy and bioavailability of natural products like the Kampo, TU‐100 (Daikenchuto) are limited if taken sporadically versus regularly. For TU‐100, this appears to involve the progressive selection of gut microbiota that increases the metabolic capacity for TU‐100 and its bioavailability. Our studies further show that this process of “self‐reinforcing” xenobiotic metabolism is selective for the ginseng component of TU‐100 and not it for its pepper and ginger counterparts. Our findings underscore a unique mechanism for enhancing drug metabolism and bioavailability that has direct clinical and practical implications.

Long term, but not acute, dietary TU‐100 increases abundance of a number of bacteria that produce short‐chain fatty acids, including butyrate, propionate, and acetate. Short‐chain fatty acids have potent immunomodulatory activities on intestinal dendritic cells and lymphocytes as shown by the enhancement of gut T‐regulatory cell development with mixtures or specific species of *Clostridiales* that decreased colitis and systemic IgE responses in an experimental animal models (Thangaraju et al. 2009; Berdnt et al. 2012; Furusawa et al. 2013; Chang et al. 2014; Singh et al. 2014).

Our studies demonstrate that dietary TU‐100 speci‐fically increases the metabolic capacity for ginsenos‐ide metabolism. Many ginsenosides are metabolized by glucosidases and glycosidases expressed by many bacteria of the intestinal tract. We propose that chronic ingestion of TU‐100 increases expression of metabolizing enzymes and increased abundance of the bacteria that express these enzymes, resulting in greater bioavailability. The observed increases in compound K, a bioactive ginsenoside Rb1 metabolite, with sustained TU‐100 consumption demonstrates this effect.

Dietary products, through changes in the intestinal microbiome, may also promote microbial selection that has other effects. Notable for TU‐100 is the increase in *Lactococcus lactis* that produces a number of bacteriocins (Sablon et al. 2000). A number of *L. lactis* strains used as probiotics have been studied for their antiviral (Maruo et al. 2012), anti‐inflammatory (Nishitani et al. 2009; Luerce et al. 2014), antimicrobial (Sikorska and Smoragiewicz 2013; Nami et al. 2014), and antiallergy (Rutten et al. 2011; Yoshida et al. 2011) and immunomodulatory effects that may be due to secreted metabolites as well as structural components. These additional actions of TU‐100 induced changes in gut microbiota can therefore be exploited for other clinical and biological applications.

The therapeutic effects of ginseng, as well as ginger and sanshools, are diverse and include vascular, cardiac, antineoplastic, and anti‐inflammatory actions (Rimar et al. 1996; Kwan et al. 2004; Ghayur and Gilani 2005; Kim et al. 2009; Song et al. 2009; Kono et al. 2011; Shin et al. 2013; Ueno et al. 2014). Some of these actions may relate to the antioxidant properties of ginseng and ginger also possesses anti‐inflammatory actions (Kitts et al. 2000; Hu and Kitts 2001; Masuda et al. 2004; Kikuzaki and Nakatani 1993; Kim et al. 2011). Both ginseng and ginger may not only have antineoplastic actions through their anti‐inflammatory actions, but also both inhibit MAP kinase activation that plays a role in cancer development (Ueno et al. 2014). The present study demonstrates that microbial actions of TU‐100 or ginseng and likely many other compounds may be multifactorial, including direct actions on cells, antioxidant actions, and also intestinal microbiome changes. The microbiome‐dependent effect is also multifactorial. By altering the microbiome, absorption and bioavailability may change, as is demonstrated in the present studies for ginsenoside saponin Rb1. Second, the altered microbiome may produce themselves altered levels of metabolites, as is shown in the present studies for butyrate that may have beneficial therapeutic effects.

In conclusion, chronic, but not acute, dietary TU‐100 reshapes the intestinal microbiome resulting in a number of effects. The bacterial‐mediated effects include increased short‐chain fatty acid production, increased ginsenoside conversion, and antimicrobial. Elucidation of the relation of TU‐100 and the microbiome effects can be used to establish and optimize the medical effects of TU‐100. Finally, the application of these principles can be used to design best practices for the use of KAMPO and other natural medicines.

## Disclosure

None declared.

## Supporting information


**Figure S1.** Representative metabolic pathways of ginseng ingredients.

**Figure S2.** Plasma concentrations of Rb1 and CK.

**Table S1**. The list of fecal bacterial genera analyzed in the present analysis. The difference between the mice fed with diet without TU‐100, and those with TU‐100 diet was analyzed by unpaired *t* test and the *P* values were shown. ND, not detected.
